# Supplementary material for: Dalbavancin as Suppressive Therapy for Implant-Associated Osteoarticular Infections
Source: Antibiotics (Basel). 2025 Nov 20;14(11):1171. doi: 10.3390/antibiotics14111171 (PMC12649215; doi:10.3390/antibiotics14111171)
Supplement: Supplementary file 1 [file antibiotics-14-01171-s001.zip › antibiotics-3944287-supplementary.pdf]

Supplementary Table S1. Description of SAT failures with dalbavancin.

	Characteristics	Implant	Timing	Microorganism	Surgical management	Cumulative dose (mg)	Time to failure (days)
1	Male, 60 y	Knee PJI	PIOC	*C.acnes*	None	2,500	10
2	Female, 37 y	Knee PJI	Chronic	*S. haemolyticus*	None	3,000	27
3	Female, 84 y	Knee PJI	Acute	Negative culture	One-stage exchange	4,500	60
4	Male, 78 y	Hip PJI	PIOC	*S. epidermidis*	One-stage exchange	3,000	120
5	Male, 78 y	Knee PJI	Chronic	*S. epidermidis*	None	34,000	276
6	Female, 87 y	Knee PJI	Chronic	*S. epidermidis*	DAIR	36,000	313
7	Male, 40 y	Knee PJI	Acute	*S. aureus*	DAIR	49,000	504
8	Female, 70 y	Knee PJI	Chronic	*S. epidermidis*	None	25,500	521
9	Male, 69 y	Knee PJI	Acute	*S. epidermidis*	DAIR	85,500	1053
10	Female, 90 y	Knee PJI	Chronic	*S. epidermidis*	DAIR	69,000	1308
11	Female, 94 y	Tibia implant	Chronic	*S. epidermidis*	None	42,000	576

Supplementary Table S2. Analysis of factors associated with SAT failure with dalbavancin.

Variables	No failure (32)	Failure (11)	p-value
Female	20 (76.9)	6 (23.1)	0.64
Age, years, median (IQR)	77.5 (60-87)	78 (60-87)	0.46
Comorbidities			
Diabetes	5 (71.4)	2 (28.6)	0.84
Tumor	7 (70.0)	3 (30.0)	0.72
Chronic kidney disease	4 (66.7)	2 (33.3)	0.64
Charlson index, median (IQR)	2 (0-3)	2 (0-3)	0.94
Immunosuppression	8 (80.0)	2 (20.0)	0.64
Implant type:			
Prosthesis	28 (73.7)	10 (26.3)	
Osteosynthesis	2 (66.7)	1 (33.3)	0.67
Spinal instrumentation	2 (100.0)	0	
Location*:			
Hip	11 (91.7)	1 (8.3)	
Knee	16 (64.0)	9 (36.0)	0.17
Shoulder	1 (100.0)	0	
Classification*:			
Acute	9 (75.0)	3 (25.0)	
Chronic	16 (76.2)	5 (23.8)	0.35
Hematogenous	2 (100.0)	0	
PIOC	1 (33.3)	2 (66.7)	
Fistula	12 (70.6)	5 (29.4)	0.64
Adequate surgery to cure infection	10 (76.9)	3 (23.1)	0.91
Surgical strategy:			
One-stage revision	1 (33.3)	2 (66.7)	
Osteosynthesis removal	1 (100.0)	0	0.17
DAIR	17 (81.0)	4 (19.1)	
Microbiological findings:			
No microbiological isolation	2 (66.7)	1 (33.3)	0.75
Positive cultures	30 (75.0)	10 (25.0)	
Subsequent regimens:			
500mg weekly	5 (83.3)	1 (16.7)	
500mg every 2 weeks	1 (100)	0	
1000mg weekly	3 (100)	0	
1000mg every 2 weeks	6 (75.0)	2 (25.0)	
1000mg every 3 weeks	1 (100)	0	0.29
1000mg monthly	2 (100)	0	
1500mg every 2 weeks	6 (54.6)	5 (45.5)	
1500mg every 3 weeks	0	1 (100)	
1500mg monthly	9 (90.0)	1 (10.0)	
Cumulative dalbavancin dose (mg), median (IQR)	27,750 (14,750-68,250)	34,000 (3,000-49,000)	0.32
Duration of dalbavancin (days), median (IQR)	169.5 (101.5-506)	152 (34-576)	0.41
Number of doses, median (IQR)	23.5 (12-52)	31 (2-46)	0.49
Combined therapy	4 (80.0)	1 (20.0)	0.76

*Variable related with PJI
